# Covid-19 mimicking symptoms in emergency gastrointestinal surgery cases during pandemic: A case series

**DOI:** 10.1016/j.ijscr.2020.10.064

**Published:** 2020-10-24

**Authors:** Adeodatus Yuda Handaya, Joshua Andrew, Ahmad Shafa Hanif, Aditya Rifqi Fauzi

**Affiliations:** Digestive Surgery Division, Department of Surgery, Faculty of Medicine, Universitas Gadjah Mada/Dr. Sardjito Hospital, Yogyakarta 55281, Indonesia

**Keywords:** COVID-19 pandemic, COVID-19 symptoms, Gastrointestinal emergency case, Emergency surgery, Respiratory symptoms

## Abstract

**Background:**

The COVID-19 pandemic has changed patient management in all sectors. All patients need to be examined for COVID-19, including in digestive surgery emergency cases. In this paper, we report four digestive surgery emergency cases with clinical and radiological findings similar to COVID-19.

**Case presentation:**

We report four digestive surgery emergency cases admitted with fever and cough symptoms. Case 1 is a 75-year-old male with gastric perforation and pneumonia, case 2 is a 32-year-old female with intestinal and pulmonal tuberculosis, case 3 is a 30-year-old female with acute pancreatitis with pleuritis and pleural effusion, and the last case is a 56-year-old female with rectosigmoid cancer with pulmonal metastases. All the patients underwent emergency laparotomy, were hospitalized for therapy, and discharged from the hospital. After 1-month follow-up after surgery, 1 patient had no complaints, 2 patients had surgical site infection, and 1 patient died because of ARDS due to lung metastases.

**Discussion:**

For all four cases, the surgeries were done with strict COVID-19 protocol which included patient screening, examination, laboratory assessment, rapid test screening, and RT-PCR testing. There were no intrahospital mortalities and all the patients were discharged from the hospital. Three patients were followed-up and recovered well with 2 patients having surgical site infection which recovered within a week. However, 1 patient did not show up for the scheduled follow-up and was reported dead 2 weeks after surgery because of ARDS due to lung metastases.

**Conclusions:**

Emergency surgery, especially digestive surgery cases, can be done in the COVID-19 pandemic era with strict prior screening and examination, and safety protocol.

## Highlights

• Digestive surgery emergency cases can present with COVID-19 mimicking symptoms.
• Indications of emergency surgery are the same in during pandemic compared to non-pandemic settings.
• Strict screening, examination, and protocol are necessary during pandemic.

## 1 Introduction

COVID-19 belongs to the coronavirus family which infect the respiratory system and cause symptoms such as fever, breathing difficulty, and even respiratory failure [1]. COVID-19 is spread through droplets and enters the host through mouth, nose, and eyes and mostly infects adults and older population with nearly half of all cases having comorbid medical conditions, for whom case fatality rates are elevated in these patients with co-existing diseases [2,3]. Until July, 27^th^ 2020, COVID-19 had infected more than 16 million people worldwide and more than 100,000 people in Indonesia [4].

Patients undergoing emergency surgery have higher mortality and morbidity rates than elective surgery patients. Data from the American College of Surgeons (ACS) and National Surgical Quality Improvement Program (NSQIP) showed a mortality of 14% at 30 days for emergency laparotomy patients in the US. Emergency status was a significant predictor for morbidity, serious morbidity, and mortality compared to elective surgery, adjusting for patient-related and operation-related risk factors, in hospital performance [5].

The COVID-19 pandemic has changed the ways patients are admitted and treated, including in emergency digestive surgery cases. In this paper, we aimed to report four digestive surgery emergency cases admitted with fever and cough symptoms, mimicking COVID-19. This work has been reported in line with PROCESS criteria [6].

## 2 Presentation of cases

### 2.1 Case 1

**Fig. 1:**
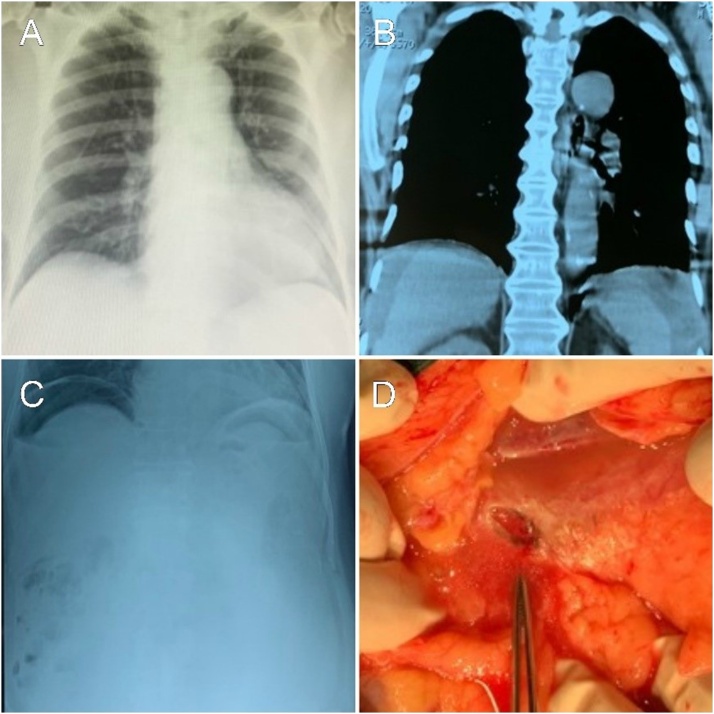
A) Chest X-ray showed bronchopneumonia, B) Chest MSCT showed emphysematous lungs, C) Abdominal X-ray showed pneumoperitoneum, and D) Intraoperative finding of gastric perforation.

A 75-year-old man was admitted to our emergency department with fever, cough, and abdominal pain. The patient had history of diabetes and gastritis diagnosed and treated by gastroenterologist, smoking history, and routinely used analgesics in a recent three consecutive years span. Ronchi and peritoneal signs were found in his physical examination. Laboratory results showed haemoglobin level of 12.5 g/dL, leucocyte count 11.6 × 10^9^/L, neutrophils 79.3%, lymphocytes 14.0%, and Neutrophils-Lymphocytes Ratio (NLR) 5.66. Both Anti-SARS-CoV-2 IgG and IgM Rapid Test showed non-reactive results. Chest X-Rays showed bronchopneumonia (Fig. 1a) and chest computed tomography (CT)-scan showed emphysematous lungs (Fig. 1b). Abdominal X-Ray showed pneumoperitoneum (Fig. 1c) and abdominal ultrasonography showed subhepatic and Morison pouch free of fluid. The patient was diagnosed with peritonitis because of hollow organ perforation with bronchopneumonia. The patient underwent exploratory laparotomy with level 3 personal protective equipment. Intraoperative finding was gastric (antrum) perforation, and omental patch was performed to close the defect (Fig. 1d). The patient was treated for two days in the intensive care unit after the surgery and moved to the general ward with stable condition. The COVID-19 PCR test with nasopharyngeal swab sample showed negative results and the pathologic finding showed helicobacter pylori infection. The patient was discharged after 7 days and followed-up weekly. After 3 weeks of follow-up the patient showed no symptoms of fever nor cough. There was minimal surgical site infection which was resolved after topical wound care.

### 2.2 Case 2

**Fig. 2:**
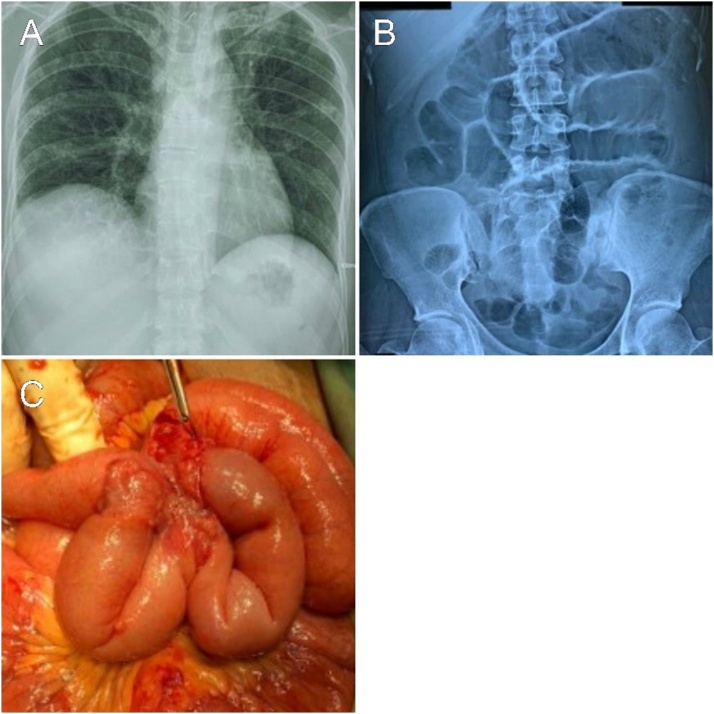
A) Chest X-ray showed atypical bronchopneumonia, B) Abdominal X-ray showed small bowel obstruction suspected ileus, and C) Intraoperative finding of multiple intestinal strictures and stenosis.

A 30-year-old woman was admitted to our emergency department with fever, cough, abdominal pain, bloating, inability to defecate, and melena. The patient had a history of Crohn’s disease with suspected colitis. Abdominal distention was found in her physical examination. Laboratory results showed Hemoglobin level of 8.7 g/dL, Leucocyte count 9.75 × 10^9^/L, Neutrophils 85.9%, Lymphocytes 7.9%, Neutrophils-Lymphocytes Ratio (NLR) 10.87, C-Reactive Protein (CRP) 171.67 mg/dL, and negative result of ANA test. Both Anti-SARS-CoV-2 IgG and IgM rapid tests showed non-reactive results. Chest X-Ray showed atypical bronchopneumonia (Fig. 2a) and chest CT scan showed bilateral pleural reaction with signs of pneumonia. Abdominal X-Ray showed ileus with small bowel obstruction suspected (Fig. 2b) and abdominal MSCT showed small intestine dilatation with sigmoid colon mass suspected. The patient was diagnosed small bowel ileus with bronchopneumonia. The patient underwent exploratory laparotomy with level 3 personal protective equipment. Intraoperative findings were multiple intestinal strictures with intestinal stenosis and ileal resection followed by end-to-end anastomosis using linear cutter was done (Fig. 2c). The COVID-19 PCR test with nasopharyngeal swab sample showed negative results and the pathologic finding showed intestinal tuberculosis. The patient was treated with streptomycin for 5 days after surgery and 6 months of anti-tuberculosis therapy were prescribed by the pulmonologist.

### 2.3 Case 3

**Fig. 3:**
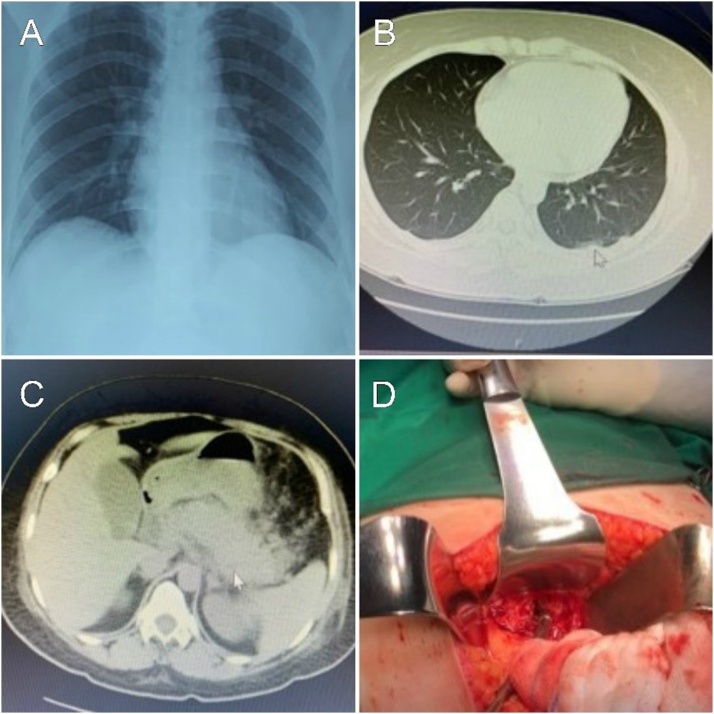
A) Chest X-ray showed normal lung and heart, B) Chest MSCT showed pleuritis, C) Abdominal MSCT showed pancreatitis necroticans, and D) Intraoperative finding of pancreatitis necroticans.

A 30-year-old woman was admitted to our emergency department with fever, cough, and upper right quadrant abdominal pain. The patient had a history of gastritis. Laboratory results showed Hemoglobin level of 12.1 g/dL, Leucocyte count 26.6 × 10^9^/L, Neutrophils 84.9%, Lymphocytes 6.6%, Neutrophils-Lymphocytes Ratio (NLR) 12.86, Amylase 193 U/L, Lipase 291 U/L, ALT 88 U/L, and AST 33 U/L. Both Anti-SARS-CoV-2 IgG and IgM rapid tests showed non-reactive results. Chest X-Ray showed normal result (Fig. 3a) and chest CT scan showed pleuritis in the lower posterior side of her left lung (Fig. 3b). Abdominal CT scan showed pancreatitis necroticans (Fig. 3c) and abdominal ultrasonography showed cholecystitis and multiple cholelithiasis. The patient was diagnosed acute pancreatitis associated with cholangitis and cholecystitis. The patient underwent exploratory laparotomy with level 3 personal protective equipment. Intraoperative findings were pancreatic necrosis, cholelithiasis, and appendicitis. Pancreatic debridement, cholecystectomy, and appendectomy were done (Fig. 3d). The surgery was followed by intraabdominal irrigation for 3 days and 5 days ward treatment. The COVID-19 PCR test with nasopharyngeal swab sample showed negative results and the pathologic finding showed pancreatitis necroticans. The patient was discharged and had no complaints in weekly follow-ups for 3 consecutive weeks.

### 2.4 Case 4

**Fig. 4:**
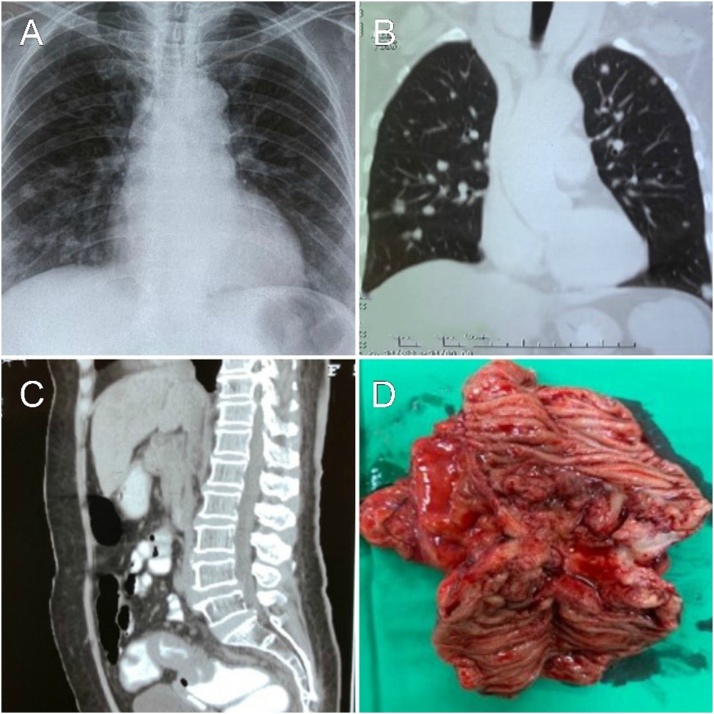
A) Chest X-ray showed multiple metastatic nodules, B) Chest MSCT showed multiple metastatic nodules, C) Abdominal MSCT showed sigmoid tumour, and D) Intraoperative finding of rectosigmoid tumor.

A 56-year-old woman was admitted to our emergency department with fever, cough, abdominal pain, bloating, and inability to defecate. The patient had history of gastritis, haemorrhoids, and previous endoscopy examination showed a rectal tumor. Abdominal distention was found in her physical examination. Laboratory results showed Hemoglobin level of 10.6 g/dL, Leucocyte count 8.4 × 10^9^/L, Neutrophils 63.6%, Lymphocytes 25.1%, Neutrophils-Lymphocytes Ratio (NLR) 2.53, and carcinoembryonic antigen (CEA) >2200 ng/mL. Both Anti-SARS-CoV-2 IgG and IgM rapid tests showed non-reactive results. Chest X-Ray (Fig. 4a) and CT scan (Fig. 4b) showed multiple metastatic nodules in both lungs without signs of bronchopneumonia or pleural effusion. Abdominal CT scan showed (Fig. 4c) 9 × 3.5 cm sigmoid tumor, posterior sigmoid lymph node enlargement, and liver metastases. The patient was diagnosed with large bowel obstruction in rectal cancer. The patient underwent exploratory laparotomy with level 3 personal protective equipment. Intraoperative finding was rectosigmoid tumor. Tumor resection and Hartmann’s procedure were done (Fig. 4d). The patient was treated 3 days in the intensive care unit after the surgery and moved to the general ward with stable condition. The COVID-19 PCR test with nasopharyngeal swab sample showed negative results and the pathologic finding showed adenocarcinoma mucoides. The patient was discharged after 10 days ward care scheduled for weekly follow-up. The patient did not show up for the scheduled follow-up and was reported dead after returning to the emergency room with ARDS due to lung metastases two weeks after discharged.

## 3 Discussion

The indications of emergency surgery are the same in this COVID-19 pandemic setting compared to non-pandemic settings. COVID-19 examination must be done as soon as possible, but the decision of surgery must be timely done in emergency cases, whether the COVID-19 test result is available or not [7].

There are some considerations in doing emergency surgery in COVID-19 pandemic settings. The virus can be transmitted in aerosol which increases the risk of infection to the anesthetist in performing intubation. Biological fluid transmission should also be considered in choosing the surgery method. Laparoscopy has been considered a good way to minimize this risk because of the closed abdominal space, however there are incidents of gas leakage in trocar removal so the laparotomy with extensive drainage is considered safer. However, the maintenance of gas pressure and flow to minimum level and the use of filter system or closed circuits to prevent uncontrolled release of pneumoperitoneum should be done. The use of energy and electrical instruments also should be maintained to the lowest energy level to avoid unnecessary production of smoke/aerosol and smoke aspirator/evacuator should be used in both laparoscopic and open surgical procedures [8,10].

To ensure the safety and minimalize the infection risk to medical personnel, the surgery should be done in an isolated closed-door surgery room. However, another consideration is duration as the surgery duration is equal to the longer exposure risk. To ensure the shortest possible time and optimize the surgical result, it would be best if the surgery is done by the minimum number of most experienced surgeons and surgical team available who have received training in surgical safety and protocol in emergency situations [8,9,11].

We report four cases of emergency digestive surgery cases with COVID-19 symptoms of cough and fever. Case 1 was a 75-year-old male with gastric perforation, case 2 was a 32-year-old woman with abdominal and pulmonary tuberculosis, case 3 was a 30-year-old woman with acute pancreatitis and minimal pleural effusion, and case 4 was a 56-year-old female rectosigmoid cancer patient with lung metastasis. All patients underwent laparotomy procedure and COVID-19 screenings were done prior to surgeries. All patients received definitive therapies according to their diseases and were examined for COVID-19 with RT-PCR test with negative results.

Emergency surgery in digestive surgery cases with COVID-19 signs and symptoms can be done with strict examination, assessment, and protocol. Recommendations for operating room personnel to minimize infection risk in COVID-19 confirmed or suspected cases are as follows [12]: (1) Use of personal protective equipment (N95 respirator and suit), as well as hand hygiene and proper equipment’s removal and disposal according to the local policy, (2) Minimalize aerosol and droplet transmission in some procedures such as endotracheal intubation, tracheostomy, gastrointestinal endoscopy, and evacuation of pneumoperitoneum and aspiration of body fluids during laparoscopic procedures, by minimalizing personnel in operating room until anesthesia induction and intubation are done, use of a negative pressure operating room if available, use of a smoke evacuator if electrocautery is needed, and consider avoiding laparoscopic and tracheostomy procedure, (3) After surgery, patient transport should be done with minimum number of personnel and protective equipment should not be the same as the one used in operating room, and (4) Personnel should wear scrub clothes upon arrival at the hospital, shower and change to a clean scrub clothes or home clothes after procedure, and maintain physical distancing and frequent hand washing.

## 4 Conclusions

This present study demonstrates emergency digestive surgery cases which have to be done to reduce the patient suffering and mortality. Emergency surgery cases with COVID-19 signs and symptoms are safe if performed with strict screening, examination, and protocol. Accompanying conditions and advanced state of cancer resulted in one death due to ARDS 1 month after surgery in case 4. Further prospective study with larger samples and longer follow-up periods are needed to confirm our findings and support making stricter clinical guidelines.

## Declaration of Competing Interest

The authors report no declarations of interest.

## Sources of funding

The authors declare that this study had no funding source.

## Ethical approval

The informed consent form was declared that patient data or samples will be used for educational or research purposes. Our institutional review board also do not provide an ethical approval in the form of case series.

## Consent

Written informed consent was obtained from all of the patients for publication of this case report and accompanying images. A copy of the written consent is available for review by the Editor-in-Chief of this journal on request

## Author contribution

Adeodatus Yuda Handaya conceived the study and approved the final draft. Aditya Rifqi Fauzi, Ahmad Shafa Hanif, and Joshua Andrew drafted the manuscript and critically revised the manuscript for important intellectual content. Adeodatus Yuda Handaya, Aditya Rifqi Fauzi, Ahmad Shafa Hanif, and Joshua Andrew facilitated all project-related tasks.

## Registration of research studies

ClinicalTrials.gov.

NCT04450277.

## Guarantor

Adeodatus Yuda Handaya.

## Provenance and peer review

Not commissioned, externally peer-reviewed.

## CRediT authorship contribution statement

**Adeodatus Yuda Handaya:** Conceptualization, Methodology, Resources, Writing - original draft. **Joshua Andrew:** Writing - review & editing, Resources, Validation. **Ahmad Shafa Hanif:** Writing - review & editing, Resources, Validation. **Aditya Rifqi Fauzi:** Writing - review & editing, Resources, Validation.
